# Supplementary material for: Anticancer Plant Secondary Metabolites Evicting Linker Histone H1.2 from Chromatin Activate Type I Interferon Signaling
Source: Int J Mol Sci. 2025 Jan 4;26(1):375. doi: 10.3390/ijms26010375 (PMC11722331; doi:10.3390/ijms26010375)
Supplement: Supplementary file 1 [file ijms-26-00375-s001.zip › revised/ijms-3293243_revised.pdf]

Article

Anticancer Plant Secondary Metabolites Evicting Linker Histone H1.2 from chromatin Activate Type I Interferon Signaling

Olga Vlasova[1,*], Irina Antonova[1], Khamis Magomedova[1], Alena Osipova[1,2], Polina Shtompel[1], Anna Borunova[1], Tatiana Zabotina[1], Gennady Belitsky[1], Irina Budunova[4], Albert Jordan[5], Kirill Kirsanov[1,3], Marianna Yakubovskaya[1,*]

[1] N.N. Blokhin National Medical Research Center of Oncology, Ministry of Health of Russia 24 Kashirskoe Shosse, Moscow 115522, Russia
[2] SBHI Moscow Clinical Scientific Center Named after Loginov MHD, 111123 Moscow, Russia
[3] Peoples' Friendship University of Russia Miklukho-Maklaya St., Moscow 117198, Russia
[4] Department of Dermatology, Northwestern University, Chicago, IL 60611, USA
[5] Institut de Biologia Molecular de Barcelona (IBMB-CSIC), Barcelona, Catalonia 08028, Spain
* Correspondence: OV olya_vlasov@mail.ru; MY mgyakubovskaya@mail.ru; Tel.: +7 925 6761167

Abstract: Previously we discovered that among 15 DNA-binding plant secondary metabolites (PSMs) possessing anticancer activity, 11 compounds cause depletion of the chromatin-bound linker histones H1.2 and/or H1.4. Chromatin remodeling or multiH1 knocking-down is known to promote the upregulation of repetitive elements, ultimately triggering an interferon response. Herein, using HeLa cells and applying fluorescent reporter assay with flow cytometry, immunofluorescence staining and quantitative RT-PCR, we studied effects of PSMs both evicting linker histones from chromatin and not influencing their location in nucleus. We found that (1) 8 PSMs, evicting linker histone H1.2 from chromatin, activated significantly type I Interferon (IFN) signaling pathway and out of these compounds resveratrol, berberine, genistein, delphinidin, naringenin and curcumin also caused LINE1 expression. Fisetin and quercetin, which also induced linker histone H1.2 eviction from chromatin, significantly activated only type I IFN signaling, but not LINE1 expression; (2) curcumin, sanguinarine and kaempferol, causing significant depletion of the chromatin-bound linker histone H1.4 but not significantly influencing H1.2 presence in chromatin, activate type I IFN signaling less intensively without any changes of LINE1 expression; (3) four PSMs, which did not cause linker histone eviction, displayed neither IFN signaling activation nor enhancement of LINE1 expression. Thus, we have shown for the first time that chromatin destabilization observed by depletion of chromatin-bound linker histone H1.2 caused by anticancer DNA-binding PSMs is accompanied by enhancement of type I IFN signaling, and LINE1 expression often impacts this activation.

Keywords: plant secondary metabolites; plant polyphenols; phytochemicals; DNA-binding compounds; anticancer effects; chromatin structure; linker histone eviction; type I interferon signaling; LINE1 transcription; double-stranded DNA ends

Citation: To be added by editorial staff during production.

Academic Editor: Firstname Lastname

Received: date
Revised: date
Accepted: date
Published: date

Copyright: © 2024 by the authors. Submitted for possible open access publication under the terms and conditions of the Creative Commons Attribution (CC BY) license (https://creativecommons.org/licenses/by/4.0/).

1. Introduction

A whole number of plant secondary metabolites (PSMs) or phytochemicals, largely consisting of polyphenols, have been shown to possess anticancer activity against chemically induced animal tumors of various types [1-4]. They reduce the incidence and multiplicity of benign and malignant tumors induced in rodents in colon by 1,2-dimethylhydrazine or azoxymethane, in breast and ovary by 7,12-dimethylbenz(a)anthracene and in breast by N-methyl-N-nitrosourea. Mechanistic data, obtained on human cancer cells growing *ex vivo* and cultured *in vitro*, confirmed antiproliferative, proapoptotic, anti-inflammatory and immunomodulatory effects of PSMs [5-8]. Chemopreventive effects of genistein, resveratrol, berberine and some other PSMs were shown in a number of clinical trials [2, 9-11].

Anticancer PSMs, characterized by the presence of aromatic rings with hydroxyl and other substituents, can bind various molecular targets in cells, such as receptors, enzymes of xenobiotic metabolism and epigenetic regulation of transcription, components of signaling pathways, enzymes of DNA repair and metabolism [12-15]. A wide range of PSM targets makes it extremely difficult to analyze consequences of different peculiar interactions, which impact the integral result of PSM action. Having unique structure, every PSM is characterized by its own spectrum of targets, however, most anticancer PSMs possess affinity to DNA. PSMs interact with DNA via van der Waals, ionic, and hydrogen bonds without forming covalent bonds, which explains why they are not genotoxic. Intercalation into DNA helix was shown for apigenin, delphinidin, fisetin, epigallocatechin-3-gallate (EGCG), genistein, naringenin, quercetin, resveratrol and sanguinarine [16-21]. Curcumin and sanguinarine interact with DNA as minor groove binders [16, 22]. G-quadruplex binding and stabilization were shown for berberine, curcumin, EGCG, fisetin, kaempferol, quercetin, sanguinarine [23-29]. Formation of DNA-PSM complexes can affect the spatial characteristics of DNA duplex, its flexibility and physicochemical properties, as well as its ability to form various alternative DNA structures [19, 30]. PSMs can cover up DNA sites, which are recognized by enzymes of DNA repair, packaging, epigenetic regulation, transcription and replication analogously to minor grove ligands preventing interaction of PARP1 with DNA duplex [31].

Previously we demonstrated that most of DNA-binding PSMs (11 compounds out of analyzed 15 PSMs with anticancer activity) cause eviction of linker histones H1.2 and H1.4 from chromatin [32]. Noteworthy, in multiH1 knocked-down cells, chromatin opening promotes the upregulation of repetitive elements, ultimately triggering an interferon (IFN) response [33]. In particular, Izquierdo-Bouldstridge et al. demonstrated that histones H1.2 and/or H1.4 are involved in the expression control of transposable elements (TEs). In general, H1 linker histones are enriched in the constitutive heterochromatin with silent repetitive elements LINEs, SINEs, and repeats containing endogenous retroviruses [34, 35]. Chromatin-related effects of DNA-binding small molecules started to be investigated about 10 years ago, demonstrating histone eviction from chromatin by anticancer agents from anthracycline group and anticancer drug Curaxin CBL0137 [36; 37]. Then anticancer activity of Curaxin CBL0137 was shown to be decreased in mice with knocked out IFNAR1, responsible for type I IFN signaling activation, and it was reduced in immune deficient SCID mice when compared to immune competent mice [38, 39]. Curaxin CBL0137 ability to induce type I IFN signaling was explained by enhanced transcription of repetitive heterochromatin elements as double-stranded RNA induce this signaling pathway.

Compounds that interact with DNA without causing DNA alterations, but induce changes in chromatin structure make constitutive heterochromatin accessible to the transcriptional machinery. Divergent transcription of centromeric and pericentromeric repeats leads to the accumulation of double-stranded RNA. It is recognized by cytoplasmic nucleic acid sensitive receptors and activates the IFN response [38]. It should be also noted that chromatin remodeling, caused by ATRX protein, was also demonstrated to activate type I IFN signaling [40].

Activation of this signaling pathway is realized by IFNs, a broad class of cytokines, representing key modulators of the immune response. These cytokines with potent antiviral and growth-inhibitory effects play critical roles in the first line of defense against infections and homeostatic disorders during cancer pathogenesis [41; 42]. IFN signaling activation was described in several studies devoted to effects of some PSMs. In particular, IFN activation was observed when cells were treated with resveratrol [43, 44], berberine [45, 42], fisetin [46], naringenin [47, 48], sanguinarine [49], quercetin [50]. All these studies were performed using single PSMs and different cancer cell lines which makes it difficult to compare their effects, and they do not show possible mechanisms of IFN activation. However, these data and our previously obtained results concerning PSM influence on

linker histone location in cell nuclei provide a good basis for clarifying the question of whether PSM-induced chromatin destabilization is accompanied by IFN activation. This clarification should both expand our understanding of molecular effects induced by anticancer PSMs and reveal cell response on chromatin destabilization caused by different DNA-binding small molecules. The latter may serve as the basis for the development of new non-genotoxic chemopreventive and anticancer drugs targeting chromatin structure and function [30].

Thus, we propose that PSMs bind DNA and cause some distortions of the helix. It is followed both by linker histone eviction from chromatin and type I IFN signaling activation. As linker histone eviction from chromatin induce transcription of silent repetitive elements, it may impact type I IFN signaling activation. The aims of the present study include analyzing the influence of 15 anticancer DNA-binding PSMs on IFN-signaling activity, on the patterns of IFN-responsive genes and on transcription of repetitive non-coding DNA. At last, the main goal of our study was to compare the data obtained with the previously described abilities of PSMs to cause linker histones H1.2 and H1.4 evictions from chromatin [32]. We chose HeLa and T47D cells as the object of our study as previously it was on these cells that we observed linker histones H1.2 and H1.4 evictions from chromatin under PSM treatment.

2. ***Results***

2.1. Type I interferon signaling activation by DNA-binding PSMs

In our study we employed two alternative approaches for assessment of IFN signaling activity in HeLa cells. Firstly, we used flow cytometry and the reporter assay that revealed IFN response through the activation of a consensus ISRE driving mCherry red fluorescent protein transgene expression in HeLa TI ISRE-mCherry cells. Previously we demonstrated that this approach of IFN response assessment is highly sensitive [51]. Secondly, we used Human Signal Transduction Pathway Finder RT2Profiler PCR Array (HSTPF, Qiagen, PARN-014Z) to analyze the changes in the expression pattern of 84 INF-responsive genes. Dose-dependence for PSM toxic effects in HeLa cells was described in our previous publications, and based on those data we chose non-toxic and IC20 (leaving more than 80% of cells alive) concentrations of PSMs for our study (Table S1) [32, 53]. Untreated cells and cells treated with the solvents were used as negative controls, while cells treated with IFN-α were used as the positive control. As the main goal of our study was to compare PSM effects on IFN activation and LINE1 expression with their ability to cause linker histones H1.2 and H1.4 evictions from chromatin described previously [32], PSM order for the effect presentation in all the figures was as follows: 1-8 – PSMs causing intensive linker histones eviction from chromatin (mainly H1.2, but accompanied with H1.4), 9-11 – PSMs causing significant H1.4 eviction from chromatin, but insignificant depletion of chromatin-bound H1.2, and 12-15 – PSMs unable to cause both H1.2 and H1.4 eviction from chromatin.

2.1.1. PSM influence on reporter mCherry expression driven by IFN-sensitive response element

Using the reporter assays and flow cytometry, we observed very intensive IFN response almost in all HeLa TI ISRE-mCherry cells after 24 h treatment with berberine, curcumin, fisetin and naringenin, resveratrol, while IFN-α treatment activated mCherry expression in 99.0% of cells (Fig 1). Significantly increased proportions of the cells expressing mCherry was also observed after cell treatment with 4 PSMs, in particular, for genistein – by 70.5%, for sanguinarine – by 60.3%, for quercetin – by 33.5% and delphinidin – by 22.5%. We did not observe significant increases in proportions of cells expressing mCherry after the treatment with apigenin, coumarin, ginsenoside Rb1, and thymoquinone, EGCG, kaempferol. Significant increases of mCherry mean fluorescence intensity (MFI) were observed in HeLa TI ISRE-mCherry cells treated with 5 PSMs, although they

were less intensive compared to the MFI in cells treated with IFN. In particular, IFN caused MFI to increase by 13.3 times, while naringenin – by 12.7 times, fisetin – by 11.8 times, curcumin and resveratrol – by 9.3 times and berberine – by 8.9 times (Fig. 1B).

For PSMs, which caused significant increase in the proportion of cells expressing mCherry after 24 h treatment, we also analyzed dynamics of the changes after 1, 6 and 24 h PSM treatment (Fig. 2).

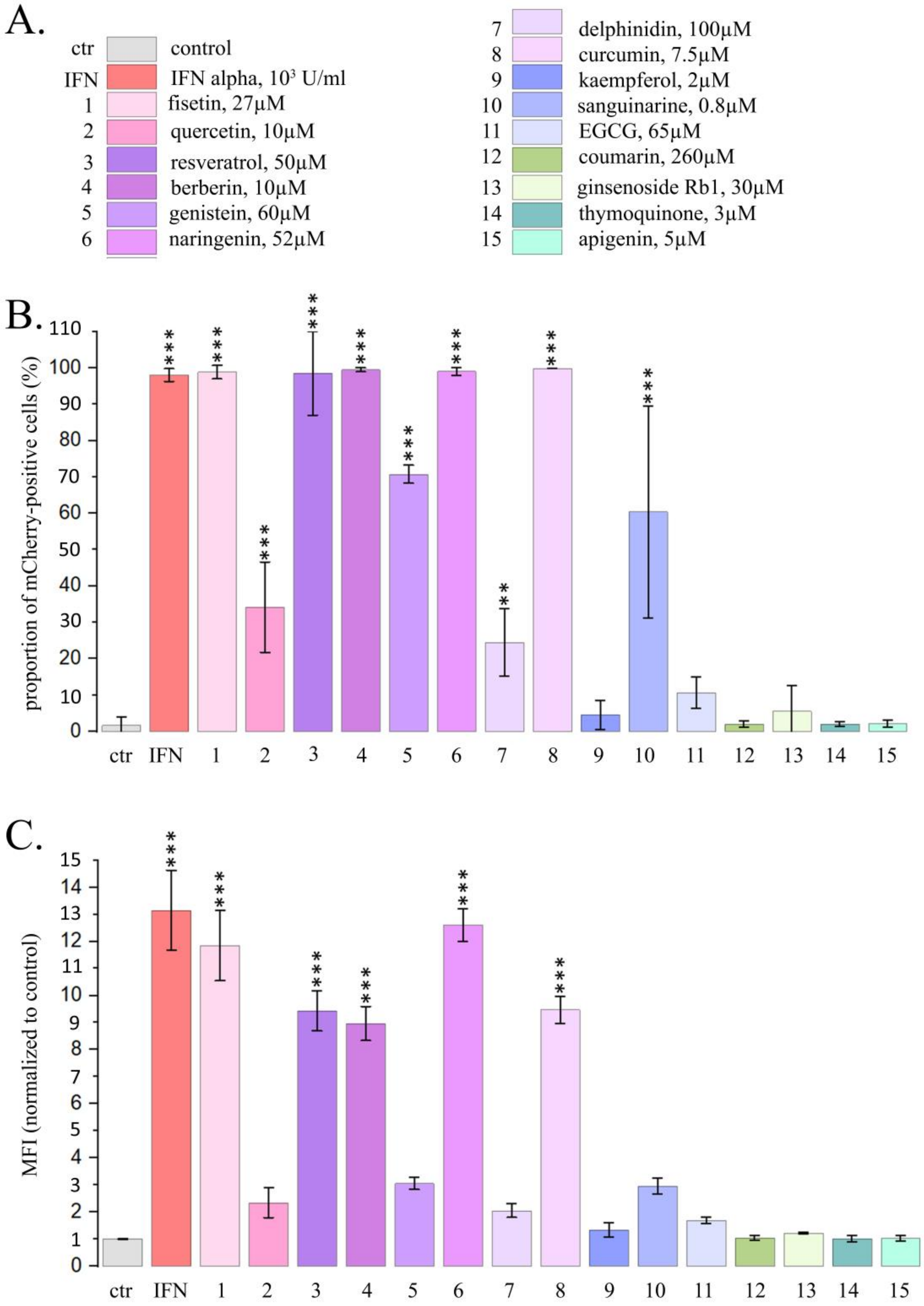

Figure 1. Flow cytometry data for the expression of mCherry driven by IFN sensitive responsive element (ISRE) in HeLa TI ISRE-mCherry cells after PSM treatment for 24 h. A. Color-numeric designation of PSMs and their non-toxic concentrations. Ctr- control; IFN- IFN-α, 103

U/ml; 1- fisetin, 27μM; 2- quercetin, 10μM; 3- resveratrol, 50μM; 4- berberine, 10μM; 5-genistein, 60μM; 6-naringenin, 52μM; 7-delphinidin, 100μM; 8- curcumin, 7.5μM; 9-kaempferol, 2μM; 10- sanguinarine, 0.8μM; 11- EGCG, 65μM; 12- coumarin, 260μM; 13- ginsenoside Rb1, 30μM; 14- thymoquinone, 3μM; 15- apigenin, 5μM. This color-number legend is used in all figures. B. Proportions of the cells exWpressing mCherry. C. Mean fluorescence intensity of mCherry per cell (normalized to control). The data are presented as an average value ± SD. Significance of the differences between control untreated cells and PSM treated cells was determined using ANOVA test and Dunnett's post hoc test: significant difference, *— $p < 0.05$, **— $p < 0.01$, ***— $p < 0.001$, ****— $p < 0.0001$.

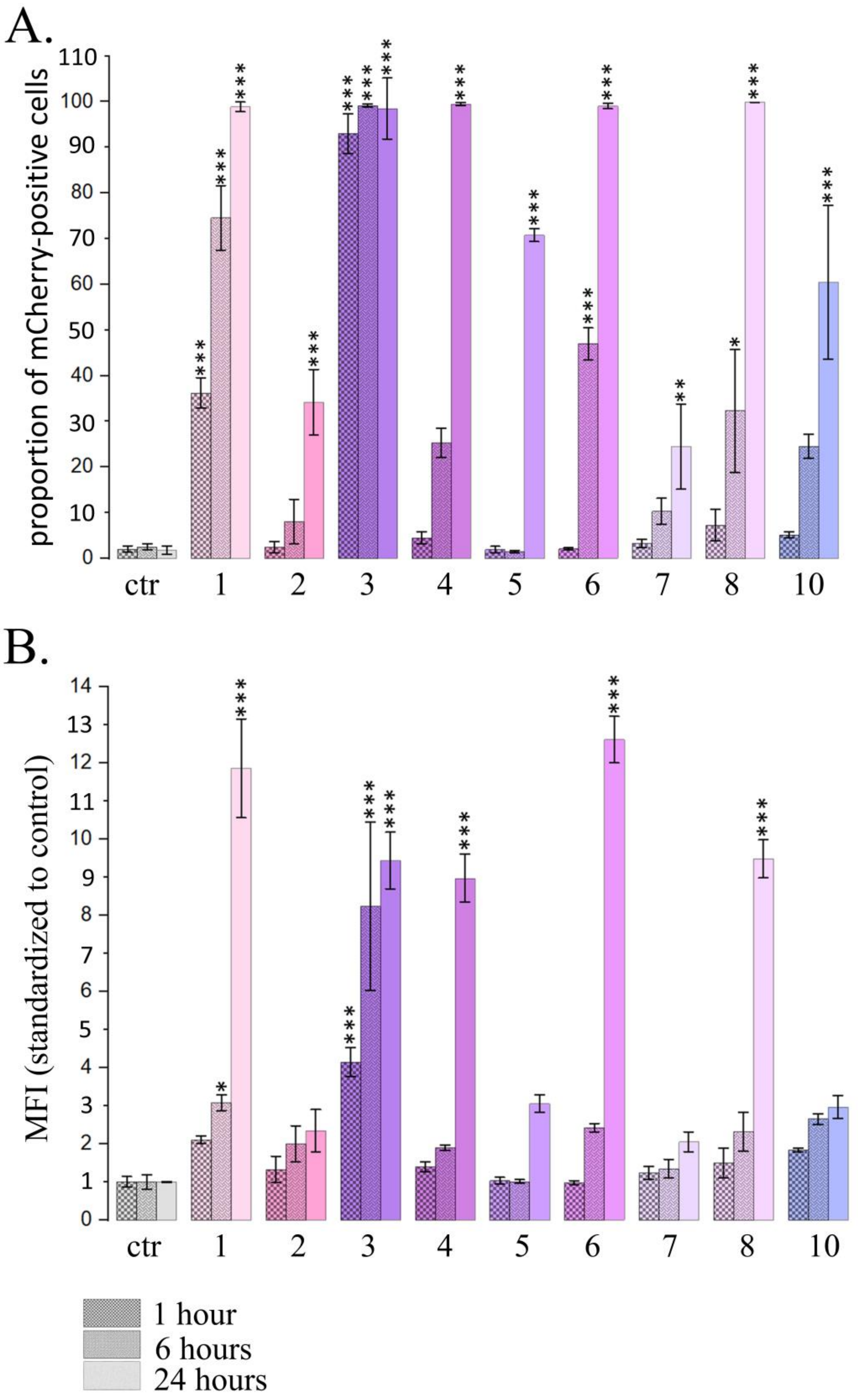

Figure 2. Flow cytometry data for the expression of mCherry driven by IFN sensitive responsive element (ISRE) in HeLa TI ISRE-mCherry cells after PSMs treatment for 1, 6 and 24 h. Ctr- control; 1- fisetin, 27μM; 2- quer-cetin, 10μM; 3- resveratrol, 50μM; 4- berberine, 10μM; 5- genistein, 60μM; 6-naringenin, 52μM; 7-delphinidin, 100μM; 8- curcumin, 7.5μM; 10- sanguinarine, 0.8μM. A. Proportions of the cells expressing mCherry. B. Mean fluorescence intensity of mCherry per cell (normalized to control). The data are presented as m±

SD. Significance of the differences between control untreated cells and PSM treated cells was determined using ANOVA test and Dunnett's post hoc test: significant difference, *— $p < 0.05$, **— $p < 0.01$, ***— $p < 0.001$, ****— $p < 0.0001$.

We observed significant increase both in the proportion of cells expressing mCherry and in the mean fluorescence intensity after 1 h treatment with fisetin and resveratrol, and the effects increased time-dependently after 6 h and 24 h treatment (Fig. 2). Curcumin and naringenin caused significant effect after 6 h treatment and their effects were enhanced after 24 h treatment. Berberine, genistein, sanguinarine and delphinidin caused significant effects only after 24 h treatment. MFI increases after the treatment with curcumin, berberine and naringenin were observed only after 24 h treatment.

Thus using reporter assay and flow cytometry we demonstrated activation of type I IFN signaling pathway after treating HeLa TI ISRE-mCherry cells for 24 hours with 9 PSMs. Two of them (resveratrol and fisetin) caused significant increase even after 1 h treatment.

2.1.2. Influence of PSMs on the expression pattern of IFN-responsive genes

Expression pattern of IFN-responsive genes was analyzed after 24 h treatment with 15 PSMs (non-toxic concentrations) and IFN-α (103UI/ml) as the positive control and using Human Signal Transduction Pathway Finder RT2Profiler PCR Array (HSTPF, Qiagen, PARN-014Z) (Fig. 3).

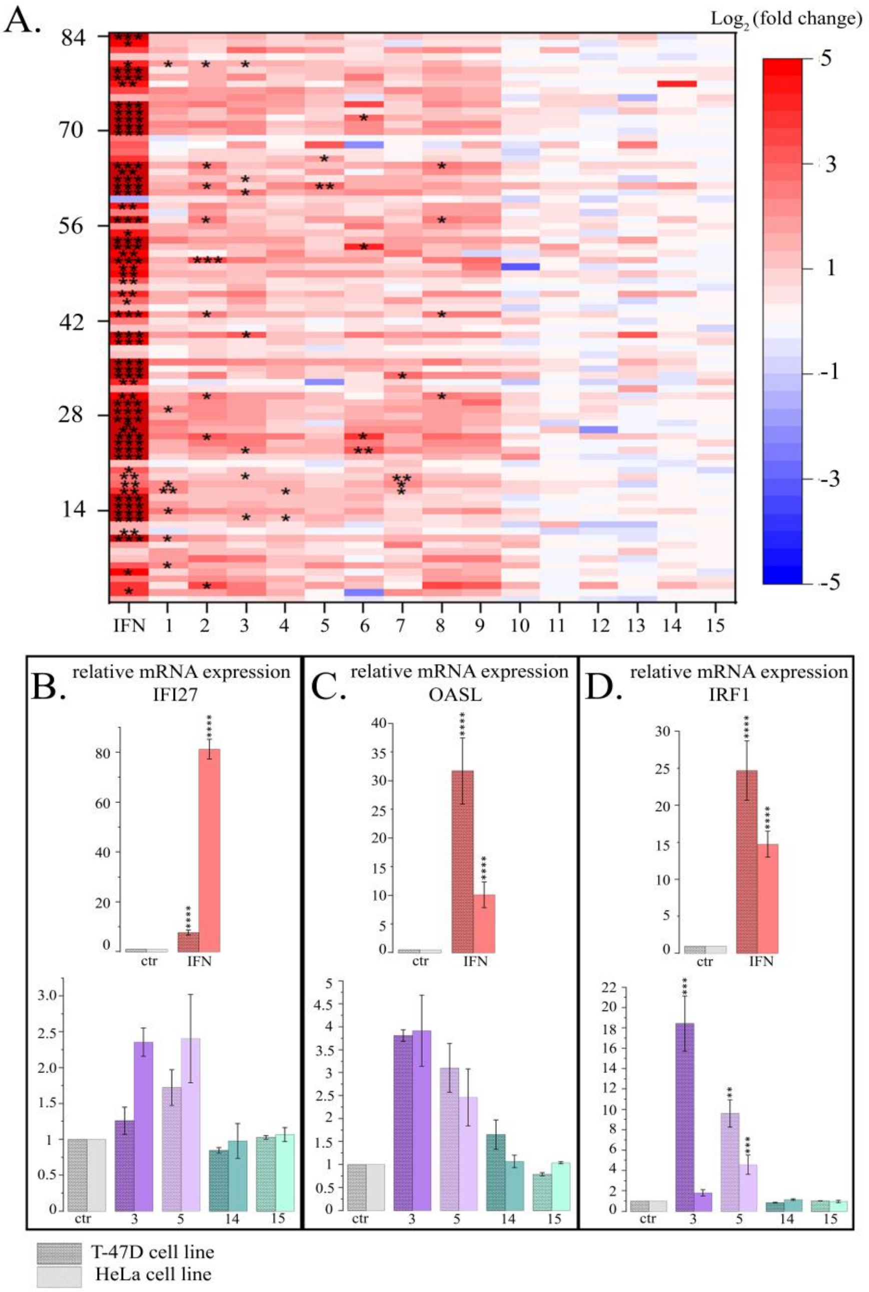

Figure 3. Influence of PSMs on gene expression of type I IFN signaling pathway. A. Pattern of gene expression of type I IFN signaling. Data presented as Log2 (fold change) for each of 84 genes (HSTPF, Qiagen, PARN-014Z). IFN- IFN-α, 103 U/ml; 1- fisetin, 27μM; 2- quercetin, 10μM; 3-resveratrol, 50μM; 4- berberine, 10μM; 5- genistein, 60μM; 6-naringenin, 52μM; 7-delphinidin, 100μM; 8- curcumin, 7.5μM; 9- kaempferol, 2μM; 10- sanguinarine, 0.8μM; 11- EGCG, 65μM; 12-coumarin, 260μM; 13- ginsenoside Rb1, 30μM; 14- thymoquinone, 3μM; 15- apigenin, 5μM. Significance of the differences between control untreated cells and PSM treated cells was determined using ANOVA test and Dunnett's post hoc test: significant difference, *— $p < 0.05$, **— $p < 0.01$, ***— $p < 0.001$, ****— $p < 0.0001$. B-D. mRNA expression of genes IFN-signaling normalized to RPL0 and HAPDH in T-47D and HeLa cell lines. Ctr- control; IFN- IFN-α, 103 U/ml; 3- resveratrol, 50μM; 5- genistein, 60μM; 14- thymoquinone, 3μM; 15- apigenin, 5μM. The data are presented as m± SD. Significance of the differences between control untreated cells and PSM treated cells was determined using ANOVA test and Dunnett's post hoc test: significant difference, *— $p < 0.05$, **— $p < 0.01$, ***— $p < 0.001$, ****— $p < 0.0001$. B. IFN-responsive gene IFI27. C. IFN-responsive gene OASL. D. IFN regulatory factor IRF1.

Hela cells 24 h treatment with IFN-α, which was used as a positive control, caused significant expression increase of all the IFN-mediated genes analyzed. Analogous cell treatment with a number of PSMs, in particular, fisetin, curcumin, berberine, resveratrol, genistein, quercetin, kaempferol, naringenin and delphinidin, caused expression activation of the most genes of type I IFN signaling pathway (Fig. 3). However, significant increases were observed only for a number of individual genes: 9 genes for quercetin, 7 genes for resveratrol and fisetin, 4 genes for curcumin and naringenin, 2 genes for berberine and genistein, and 1 gene for thymoquinone. Noteworthy, in the treated cells among 84 genes of type I IFN signaling pathway the number of genes whose expression levels were more than doubled was 62 for resveratrol, 61 for quercetin, 59 for fisetin, 54 for berberine, 53 for naringenin, 52 for curcumin, 48 for genistein, 49 for kaempferol, and 46 for delphinidin. It is also worth noting that 14 genes turned out to be common for the following compounds: CXCL10, IFI27, IFNA14, IFNA16, IFNA2, IFNA4, IFNA7, IFNG, IL20RA, IL5RA, IRF8, IRGM, ISG15. We observed a larger number of type I IFN signaling pathway genes enhanced their expression when cells were treated with sanguinarine, kaempferol, and EGCG, however, these effects were not significant. We observed very low activation level or even weak inactivation for the genes of type I IFN signaling pathway for apigenin, coumarin, ginsenoside Rb1 and thymoquinone.

Thus, using Human Signal Transduction Pathway Finder RT2Profiler PCR Array we also observed activation of type I IFN signaling pathway after HeLa cell treatment with 8 of 15 studied PSMs, and 4 PSMs not causing linker histone eviction did not produce unidirectional influence (Table S).

We studied the effect of resveratrol, genistein, apigenin and thymoquinone at non-toxic concentrations on the activation of type I IFN signaling genes expression (IFN - responsive genes IFI27 and OASL, and IFN regulatory factor IRF1) in another cell line, T47D, human breast cancer cells. According to our previous data, resveratrol and genistein cause active depletion of chromatin-bound linker histones H1.2 and H1.4 both in HeLa and in T-47D cells, while apigenin and thymoquinone did not demonstrate this effect on the HeLa cells. We observed an increase in relative gene expression, which was significant for gene IRF1 in both cell lines after 24-hour treatment of cells with resveratrol and genistein, while the levels of relative expression were comparable to control ones after cell treatment with apigenin or thymoquinone. The data obtained are comparable between two cell lines, indicating a general pattern of observations (Fig. 3 B-D).

2.2. Induction of LINE1 expression by some PSMs

Influence of PSMs on LINE1 expression was studied using two alternative methods: (1) assessment by qRT-PCR of the expression levels of three LINE1 amplicons (A, B, C) and the encoded in LINE1 gene *ORF1 LINE1* of nucleic acid-binding protein, which is essential for retrotransposition of LINE-1, and (2) immunofluorescence/flow cytometry analysis of the cells with stained ORF1 LINE1 and γ-H2AX proteins.

2.2.1. Quantitative estimation of LINE1 expression level in HeLa cells treated with PSMs

The most pronounced activation of the expression of transposable LINE1 sequences was observed when HeLa cells were treated with delphinidin. It caused the expression levels of LINE1 amplicons A, B and C to enhance by 3.7; 3.8 and 3.6 times, respectively, and also the expression level of the ORF1 LINE1 gene to increase by 4.9 times (Fig. 4).

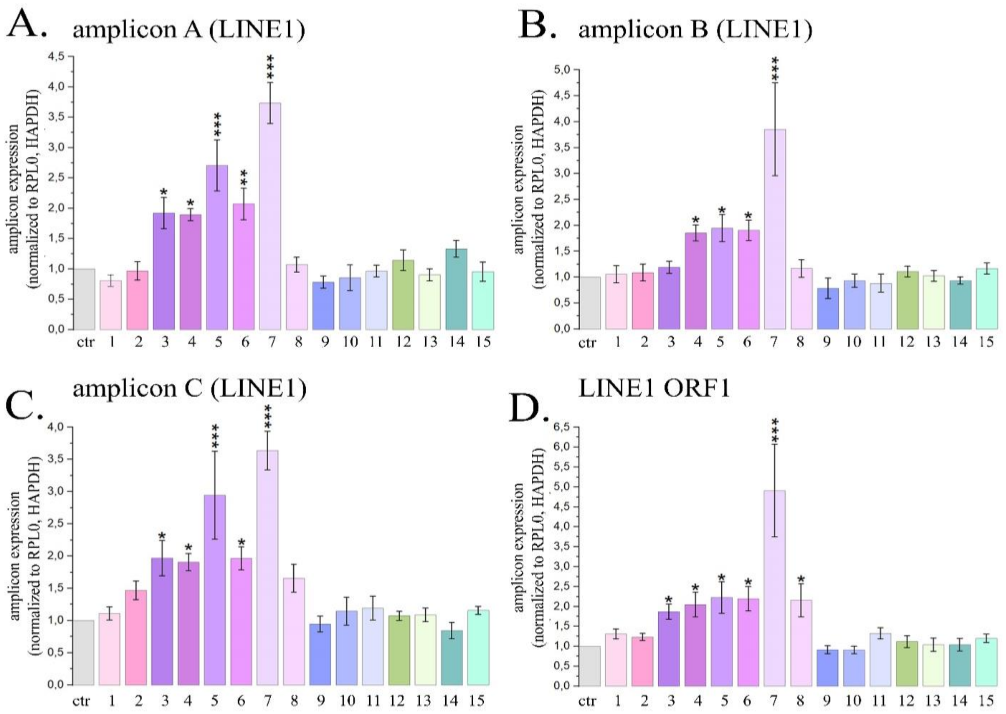

Figure 4. Expression of three LINE1 amplicons (A, B, C) and ORF1 LINE1 gene in HeLa cells treated with maximal non-toxic concentrations of PSMs for 24 h. Ctr- control; 1- fisetin, 27μM; 2-quercetin, 10μM; 3- resveratrol, 50μM; 4- berberine, 10μM; 5- genistein, 60μM; 6-naringenin, 52μM; 7-delphinidin, 100μM; 8- curcumin, 7.5μM; 9- kaempferol, 2μM; 10- sanguinarine, 0.8μM; 11-EGCG, 65μM; 12- coumarin, 260μM; 13- ginsenoside Rb1, 30μM; 14- thymoquinone, 3μM; 15- apigenin, 5μM. The data are presented as an average value ± SD. Significance of the differences between PSM treated cells and control untreated cells was determined using ANOVA test and Dunnett's post hoc test: significant difference, *— $p < 0.05$, **— $p < 0.01$, ***— $p < 0.001$, ****— $p < 0.0001$.

For resveratrol, naringenin, genistein and berberine, the average expression of LINE 1 amplicons increased by 1.7; 2.0; 2.5 and 1.9 times, and the expression of ORF1 LINE1 increased by 1.9; 2.2; 2.2 and 2.1 times, respectively. Cell treatment with curcumin was followed by a significant increase in the expression level of ORF1 LINE1 by 2.2 ± 0.4 times. The described changes in the expression levels of LINE1 amplicons and the ORF1 LINE1 gene were significant. Treatment of cells with other PSMs used in the study did not cause a statistically significant increase in the expression levels of LINE1 amplicones and/or ORF1 LINE1 gene. Thus, in this part of the study we demonstrated that 6 PSMs, in particular curcumin, berberine, delphinidin, naringenin, genistein and resveratrol, can enhance the activity of LINE1 expression.

2.2.2. Analysis of the amount of ORF1 LINE1 and γ-H2AX proteins by flow cytometry in HeLa cells treated with PSM.

For analysis of LINE1 activity we used immunofluorescent staining with antibodies to ORF1 LINE1 and γ-H2AX proteins and analyzed populations of treated and untreated cells using flow cytometry. LINE1 retrotransposition causes appearance of DNA double-stranded breaks, and therefore γ-H2AX was used as one of the markers of active retrotransposition, despite the fact that it is not specific. It is considered to be a marker of the process. We also controlled the proportion of apoptotic cells (lower than 5%) to prevent possible interference of apoptosis and LINE1 retrotransposition (Fig. 5A).

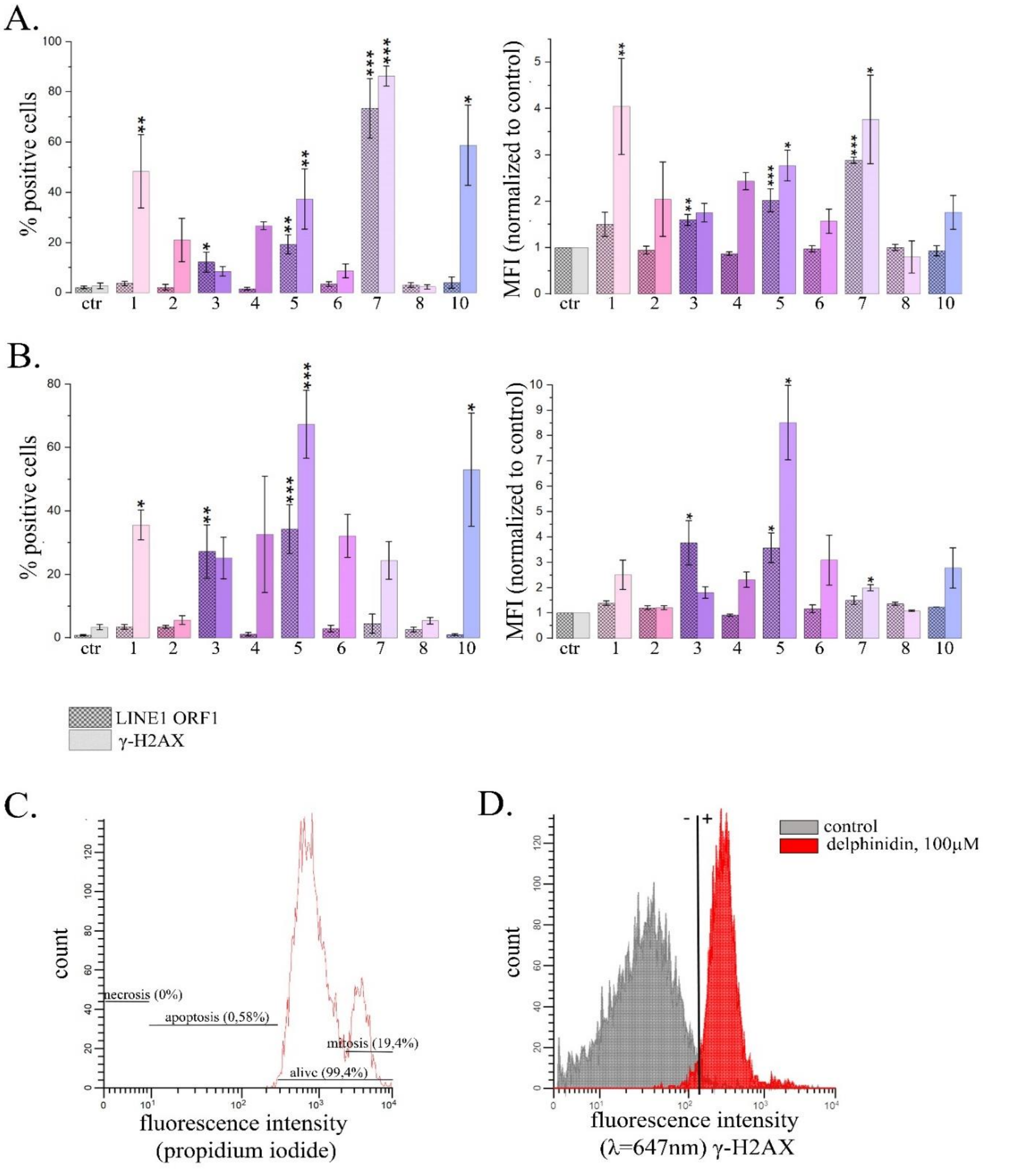

Figure 5. Flow cytometry analysis of Hela cells treated with PSMs in non-toxic concentrations and immunofluorescently stained ORF1 LINE1 or γ-H2AX. B. Example of the analysis of Hela cells with the immunofluorescently stained γ-H2AX treated with delphinidin for 24 h. A.B. The effect of PSM on the proportion of stained cells. C. 24-h PSM treatment. Ctr- control; 1- fisetin, 27μM; 2- quercetin, 10μM; 3- resveratrol, 50μM; 4- berberine, 10μM; 5- genistein, 60μM; 6-naringenin, 52μM; 7-delphinidin, 100μM; 8- curcumin, 7.5μM; 10- sanguinarine, 0.8μM. B. 72 h PSM treatment. Ctr- control; 1- fisetin, 13.5μM; 2- quercetin, 5μM; 3- resveratrol, 25μM; 4- berberine, 5μM; 5- genistein, 30μM; 6-naringenin, 26μM; 7-delphinidin, 50μM; 8- curcumin, 3,7μM; 10- sanguinarine, 0.4μM. The data are presented as an average value ± SD. Significance of the differences between control untreated cells and PSM treated cells was determined using ANOVA test and Dunnett's post hoc test: significant difference, *— $p < 0.05$, **— $p < 0.01$, ***— $p < 0.001$, ****— $p < 0.0001$. C. Measurement of proportion of apoptotic cells in the analyzed populations of fixed cells. D. Example of the analysis of Hela cells with the immunofluorescently stained γ-H2AX treated with delphinidin for 24 h.

The most pronounced effect was observed for delphinidin. 24 h treatment with delphinidin caused the cells expressing LINE1 ORF1 protein increase by 2.9 times, and the average fluorescence intensity associated with γ-H2AX appearance increased by 3.8 times. Noteworthy, with an exposure time of 72 h, delphinidin did not induce significant

changes. Genistein, on the contrary, caused a statistically significant increase in the average fluorescence intensity associated with both ORF1 LINE1 and γ-H2AX at an exposure time of 24 h by 2.0 times and 2.8 times, respectively, and after 72 h treatment at concentration of 60 μM it caused the increase by 3.6 and 8.5 times, respectively. For fisetin, only an increase in parameters associated with γ-H2AX was observed at an exposure time of 24 h. Resveratrol caused the statistically significant increase of the average fluorescence intensity associated with ORF1 LINE1 (3.7 times).

Thus, an alternative approach, although less sensitive, confirmed Influence of a number of PSMs on LINE1 expression.

3. Discussion

Over the last thirty years there has been great progress in understanding innate and adaptive immunity thanks to discovering different pattern recognition receptors (PRRs), which were shown to be associated with pathogens [54, 55].

Four major sub-families of PRRs include more than 4 hundreds of receptors, in particular, toll-like receptors (TLR), nucleotide-binding oligomerization domain – Leucin Rich Repeats-containing receptors (NLR), the retinoic acid-inducible gene 1 (RIG-1)-like receptors, and the C-type lectin receptors [56]. Current PRR discoveries allowed to present in details the genius idea of "chemical binding of exogenous substances to cell", which dominated Paul Erlich's life more than hundred years ago [54, 57]. Based on the information about immune system functioning and the role of PRR activation in immune response, P.Matzinger elaborated "the Danger theory" that immune system responses are less concerned with the self/unself origin of the antigens than with the context of its influence on tissue homeostasis [58-60]. When PRRs interact with their ligands, corresponding to danger-associated molecular patterns (DAMPs), it induces cell response. This response is in turn manifested as significant changes in cell signaling, including type I IFN signaling activation, which is considered to focus on identifying viral nucleic acids in the midst of exceedingly host-derived RNA and DNA [61].

From this point of view, the agents, which interact with DNA and change DNA-protein interactions should also form at the molecular level DAMPs, as it could influence the pattern of the transcribed sequences including both coding and non-coding DNA. Recently we have found that a number of PSMs could cause linker histone eviction from chromatin [32]. It may be proposed that PRRs recognize PSM-DNA-complexes or some other internal structures appearing after PSM-DNA complex formation.

We separated the studied PSMs into groups depending on their ability to cause eviction of linker histones from chromatin: compounds 1 to 11 in our previous experiments have this ability while compounds 12 to 15 do not (Figure 6) [32]. A more comprehensive analysis of type I IFN signaling activation pattern where we used Human Signal Transduction Pathway Finder RT2Profiler PCR Array showed results that correspond perfectly to our hypothesis: we revealed IFN signaling activation under the treatment of compounds 1-11. However, it should be pointed out that while compounds 9-11 caused significant eviction of the only linker histone H1.4, their effects were lower than the effects of compounds inducing significant eviction of H1.2.

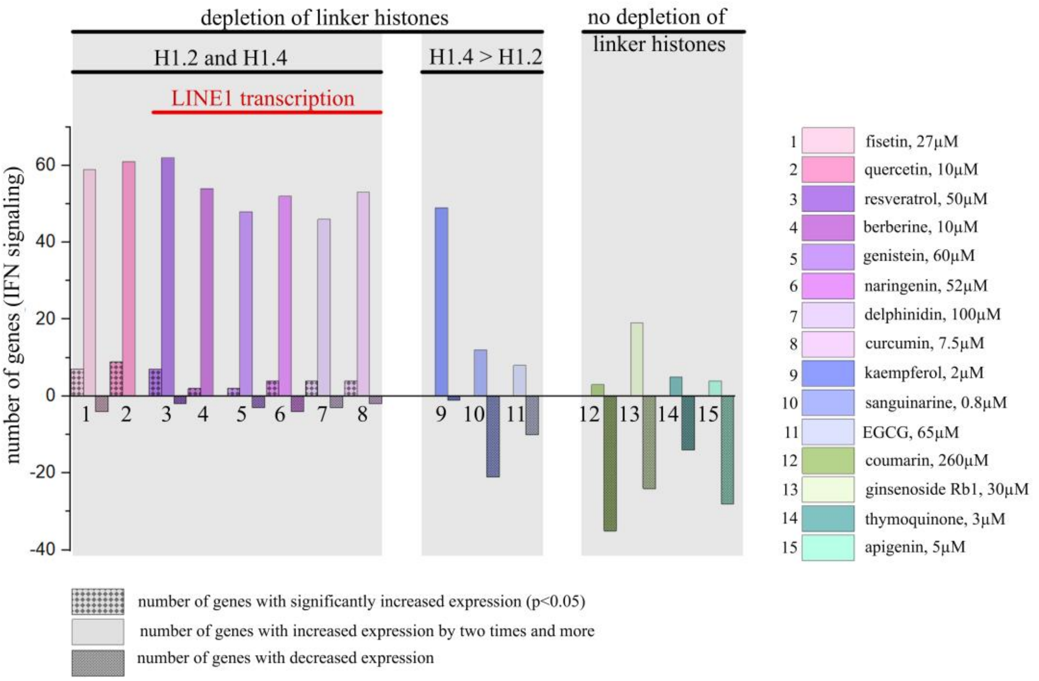

Figure 6. Activation of IFN signaling type I by PSMs (number of genes with significantly increased expression, with increased expression by two times and more and with decreased expression). PMS separated into groups depending on their ability to cause eviction of linker histones from chromatin

These data mainly correspond to the results of our ISRE-mCherry reporter analysis, and we explain some small discrepancies of the PSM effects by the fact that in this part of the study we only used ISRE to assess IFN signaling activation. The result shows that PSMs 12-15, which did not cause linker histone eviction from chromatin, are not able to induce type I IFN signaling activation that was observed in both types of experiments, when two alternative techniques of IFN signaling analysis were applied.

In regards to previously published data of type I IFN signaling activation by some PSMs, it was not studied very intensively. However, our results correspond to the published data. In particular, it was shown that quercetin and fisetin activate IFN-α in RAW 264.7 cells [62]. In the study of Lin et al. resveratrol was shown to induce TLR9 activating IFN-β signaling [63]. Activation of IFN-β signaling was also revealed in RAW264.7 and HEK293T cells after the treatment with berberine [64]. Naringenin induced IFN-α activation in U2OS cells that was demonstrated both by luciferase reporter assay and RT-PCR [65]. Sanguinarine was shown to enhance type I INF signaling in cultured monocyte-derived macrophages [66]. In the study of Ullah et al., contradictory results were published regarding genestein ability to influence type I IFN signaling: using STING competent mouse L929 cells demonstrated genistein positive effect, stably expressing an ISRE-luciferase, while the same cells cocultured with STING-deficient cGAS-overexpressing human HEK-cGASlow cells showed the opposite effect, caused by STING blocking [67]. At the same time, another study observed antiviral activity of genistein, which is considered to be the result of type I IFN signaling activation [68].

Concerning PSMs 12-15 which do not cause chromatin-bound linker histone depletion, the following data were published: thymoquinone was shown to actually decrease type I IFN signaling activity in RAW 264.7 and MCF-7 cells [69]; for genosinoside RB1 in CRFK 157 cells after 48h treatment, no activation of type I IFN signaling happened [70]; apigenin was shown to influence the inhibitory effect of IFN-α on cancer cell viability, wherein said viability is mediated by inhibition of 26S proteasome, however the effect of apigenin itself on type I IFN signaling was not analyzed [71].

Our research on the activation of IFN signaling by PSMs also revealed that PSMs 3-8, which caused significant depletion of the chromatin-bound linker histone H1.2 and H1.4, activate LINE1 expression. This observation concurs with the results of Izquierdo-Bouldstridge et al. who demonstrated that histones H1.2 and/or H1.4 participate in repression of repeats [33]. It also perfectly corresponds to a well-known fact that LINE1 expression along with other TEs stimulate type I IFN signaling [34, 72]. PSMs 9-11, which caused more significant depletion of the only chromatin-bound linker histone H1.4, induce IFN signaling less actively. It may be proposed that it is the consequences of the differential presence of H1 variants within transposable element classes and families described by Salinas-Pena et al. [34]. For instance, in T47D cells and to some extent also in HeLa cells H1.2 and H1.4 are enriched in different TEs, meaning that H1.4 is enriched in evolutionary recent SVA, Alu, L1 and LTR, while H1.2 is enriched in older TEs. Noteworthy, fisetin and quercetin (PSMs 1 and 2 in our study) did not induce LINE1 expression, although they do cause significant depletion of the histones H1.2 and H1.4. It also stands to mention that their chemical structures are very similar (Figure S1). Thus, we revealed that influence of PSMs on chromatin structure via linker histone eviction from chromatin may be accompanied by LINE1 expression enhancement, which in turn impacts type I IFN signaling activation and, consequently, impacts anticancer activity of the corresponding PSMs.

Additionally, literature search showed that peculiar direct or indirect influence on PRR-induced signaling had already been described for PSMs studied. In particular, it has already been shown that fisetin binds to TLR4, inhibits the binding of lipopolysaccharide (LPS) to the TLR4/MD2 complex and attenuates inflammatory reaction via TLR4/NLRP3 inflammasome pathway [73-76], while quercetin and resveratrol inhibit TLR4 and inflammasome activation [77-80]. Genistein [81] and berberine [45] were also demonstrated to have anti-inflammatory effects via suppression of the TLR4-mediated signaling pathway. Naringenin suppresses inflammatory responses by regulation of cell surface TLR2 functioning [47]. Delphinidin inhibits LPS-induced TLR4, MUC8, and MUC5B expression [82]. Curcumin was described to inhibit extracellular TLR 2 and 4 and intracellular TLR9 [83]. Kaempferol attenuates TLR4/NF-κB pathway activation in LPS-activated BV2 cells [84]. Sanguinarine inhibits TLR4/NF-κB pathway in H9c2 cardiomyocytes and thus attenuates LPS-induced inflammation [85], while it up-regulates expressions of endosomal TLRs [86]. EGCG was also revealed to suppress LPS-induced TLR4 activity [87, 88]. Apigenin inhibits the LPS-mediated inflammatory mediator production in keratinocytes by reducing the TLR4-dependent activation of Akt, mTOR, and NF-κB pathways [89, 90]. Thymoquinone was shown to block TLR4/NF-κB signaling pathway in microglia cells [91]. Ginesinoside Rb1 reduces TLR4 dimerization followed by inhibiting the TLR4-MyD88-NF-κB/MAPK pathways [92]. Coumarines were shown to attenuate inflammation also via TLRs [93]. Thus, anti-inflammatory effects were described for all the PSMs considered in our study, which should impact anticancer activity along with type I IFN signaling accompanying linker histone eviction from chromatin.

4. Materials and Methods

4.1. Cell Culture

The HeLa cell line was obtained from the Blokhin CRC cell collection. HeLa-TI-ISRE-mCherry cells were kindly provided by Dr. Gurova, the Department of Cell Stress Biology at Roswell Park (Buffalo, NY, USA). Preparation and maintenance of HeLa-TI-ISRE-mCherry cells, containing integrated red fluorescent protein (mCherry) gene, driven by a consensus IFN-sensitive response element (ISRE), were described previously [38]. Human breast cancer cells T47D were kindly provided by Dr. Jordan, Department of Molecular Genomics, Molecular Biology Institute of Barcelona IBMB-CSIC, Scientific Park of Barcelona, 08028 Barcelona, Catalonia, Spain. Cells were cultured in Dulbecco's Modified Eagle Medium (DMEM, C420p, PanEco, Moscow, Russia) supplemented with L-glutamine

(0.584 mg/mL) (F033E, PanEco, Moscow, Russia), penicillin (50 U/mL), and streptomycin (50 μg/mL) (A063p, PanEco, Moscow, Russia) and 10% fetal bovine serum (Biowest, S1810-500, Nuaillé, France). Cell lines were incubated at 37 °C and 5% CO2. All cell lines were validated by STR profiling and tested negative for mycoplasma.

4.2. Plant Secondary Metabolites

All of the studied compounds were obtained from Chemlight, Moscow, Russia (1). We studied apigenin (CAS 520- 36-5), (1); berberine (CAS 633-65- 8), (1); coumarin (CAS 91-64-5), (1); curcumin (CAS 458-37-7), (1); delphinidin (CAS 13270- 61-6), (1); EGCG (CAS 989-51-5), (1); fisetin (CAS 528-48-3), (1); genistein (CAS 446-72-0), (1); ginsenoside Rb1 (CAS 41753-43-9), (1); kaempferol (CAS 520-18-3), (1); naringenin (CAS 480-41-1), (1); quercetin (CAS 117-39- 5), (1); resveratrol (CAS 501-36-0), (1); sanguinarine chloride hydrate (CAS 5578-73- 4), (1); thymoquinone (CAS 490-91-5), (1).

4.3. Other Chemicals and Reagents

Curaxin CBL0137 was provided by Incuron, Inc., Russia. TRIzol™ Reagent (15596026), Moloney Murine Leukemia Virus Reverse Transcriptase (M-MLV RT) (18057018) and Random(dN)10 (SB002) were purchased from Evrogen, Moscow, Russia. dNTP mix; deionized water, nuclease-free; Taq DNA polymerase; 10X Taq Turbo Buffer; SYBR® Green dye and primers were purchased from Evrogen, Moscow, Russia. Triton X-100 (CAS 9002-93-1) was purchased from BioInnlabs, Rostov-on-Don, Russia. Dimethyl sulfoxide (DMSO, 67-68-5 | 102952), cOmplete™, Mini Protease Inhibitor Cocktail (cat. 11836153001), Phosphate-buffered saline (PBS, P4417), bovine serum albumin (CAS 9048-46-8), IFN-α A Protein, Recombinant human (P01563) were purchased from Sigma Aldrich (Merck), Bengaluru, Karnataka, India. Versene Solution (P080p), Trypsin-EDTA 0.25% solution with Hanks salts (P043p) and Phosphate-buffered saline (PBS, P4417) were purchased from PanEco, Moscow, Russia. DC™ Protein Assay Kit I (5000111EDU) was purchased from Bio-Rad (Moscow, Russia). Clarity Max™ Western ECL Substrate for Chemiluminescent Detection of Horseradish Peroxidase (HPR) Conjugates (cat. 1705062) was purchased from Helicon, Moscow, Russia. 2.5x Reaction mixture for qRT-PCR in the presence of SYBR Green I dye (M-427) was purchased from Syntol (Moscow, Russia). Antibodies LINE1-ORF1 (cat# MABC1152, 1:500) were purchased from Sigma Aldrich (Merck), Bengaluru, Karnataka, India; γ-H2AX (cat# ab26350, 1:700) and and Donkey Anti-Mouse IgG H&L (Alexa Fluor® 488, cat# ab150105; 1:1000) - from Abcam, Cambridge, UK).

4.4. Quantitative reverse transcriptase-polymerase chain reaction for Analysis of expression of LINE1 and PSM-induced Interferon Signaling

For the assay, tumor cells (HeLa) were seeded in 6-well plates (10^5 cells per well in 2 ml DMEM) and incubated with various concentrations of compounds for 24 h and IFN-α (103UI/ml) used as a positive control for the IFN Signaling analysis. Total RNA was then extracted using TRIzol™ Reagent according to the manufacturer's protocol. cDNA was synthesized using a reverse transcription reaction. Total RNA (1 μg, from both control and treated cells) was reverse transcribed using M-MLV RT reverse transcriptase and random Random(dN)10 in a reaction volume of 20 μl according to the manufacturer's protocol (Evrogen, Russia). RNA quantification was performed using NanoDrop Lite (Thermo-Scientific, Waltham, MA, USA).

For analysis of expression of amplicones LINE1 qRT-PCR was carried out in a reaction mixture containing Master Mix (0.3 mM dNTP mix (10 mM each), 3 mM MgCl2, deionized water, nuclease-free, SYBR® Green dye, 10X Taq Turbo Buffer, 0.2 U/ μl Taq DNA polymerase), 0.2 μM forward and reverse primers, 5 ng of DNA template, in accordance with the manufacturer's protocol (Evrogen, Russia). Thermal cycling conditions were as follows: initial denaturation step by heating at 95°C for 5 min, followed by 40 cycles of 15

s initial denaturation (at 95°C), 20 s at the appropriate melting temperature according to the primers, and 25 s extension at 72°C. Expression of the gene of interest was normalized to the constitutively expressed housekeeping genes RPL0 and HAPDH. The relative expression level was calculated for each sample using the 2−ΔΔCt method. All experiments were performed at least in triplicate biological replicates.

The sequences of the gene-specific primers used for qRT-PCR were as follows (Primer design from [52]):

LINE1_amplA_F: 5′GCCAAGATGGCCGAATAGGA 3′

LINE1_amplA_R: 5′AAATCACCCGTCTTCTGCGT 3′

LINE1_amplB_F: 5′CGAGATCAAACTGCAAGGCG 3′

LINE1_amplB_R: 5′CCGGCCGCTTTGTTTACCTA 3′

LINE1_amplC_F: 5′ TAAACAAAGCGGCCGGGAA 3′

LINE1_amplC_R: 5′ AGAGGTGGAGCCTACAGAGG 3′

LINE1_ORF1_F: 5′ ACCTGAAAGTGACGGGGAGA 3′

LINE1_ORF1_R: 5′CCTGCCTTGCTAGATTGGGG 3′

RPL0 F: 5′CCTTCTCCTTTGGGCTGGTCATCC A 3′

RPL0 R: 5′CAGACACTGGCAACATTGCGGACAC 3′

HAPDH F: 5′GTCTCCTCTGACTTCAACAGCG 3′

HAPDH R: 5′ACCACCCTGTTGCTGTAGCCAA 3′

The sequences of the gene-specific primers used for type I IFN signaling qRT-PCR were as follows (Primer design from [33]):

IFI27_F: 5′ TGCTCTCACCTCATCAGCAGT 3′

IFI27_R: 5′ CACAACTCCTCCAATCACAACT 3′

OASL_F: 5′ GGGACAGAGATGGCACTGAT 3′

OASL_R: 5′ AAATGCTCCTGCCTCAGAAA 3′

IRF1_F: 5′ TTTGTATCGGCCTGTGTGAATG 3′

IRF1_R: 5′ AAGCATGGCTGGGACATCA 3′

For analysis of gene expression of type I IFN signaling qRT-PCR was performed in 96-well Human Signal Transduction PathwayFinder™ RT 2 Profiler™ PCR Array plates (https://geneglobe.qiagen.com/us/product-groups/rt2-profiler-pcr-arrays/PAHS-064Z, Qiagen, PAHS-064Z, Hilden, Germany) according to the manufacturer's protocol: 95°C for 10 min, then 40 cycles of 95°C for 15 s and 60°C for 1 min. Each RT2 Profiler PCR array contains gene-specific primers for qRT-PCR assays for a carefully screened set of 84 genes, consisting of IFNs, IFN receptors, IFN regulatory factors, and IFN-responsive genes (Table 1).

Table 1. Description of the set of 84 genes analysed.

Type of gene products	Gene products	Number of genes
**IFNs (21)**	IFN-α; IFN-β; receptor ligands	5 genes
	IFN-γ; receptor ligands	1 genes
	Hematopoietin & IFN class (D200-domain) cytokine receptor ligands	10 genes
	Other IFN related genes	5
**IFN receptors (37)**	IFN-α and IFN-β receptors	2
	IFN-γ receptors	2
	Hematopoietin, IFN class (D200-domain) receptors	28
**IFN regulatory factors (9)**		9
**IFN-responsive genes (23)**	Response to virus	13*
	Transcriptional regulation	2*
	Other IFN responsive genes	8

**Note: The IFI16 gene is repeated in groups of Response to virus and transcriptional regulation.*

Expression of genes of interest was normalized to constitutively expressed housekeeping genes (ACTB, B2M, GAPDH, HPRT1, RPLP0). Relative expression levels were calculated for each sample using the $2^{-\Delta\Delta Ct}$ method using the manufacturer's software. All experiments were performed at least in triplicate biological replicates.

4.5. Analysis of ISRE-mCherry reporter activation in HeLa-TI-ISRE-mCherry cells by flow cytometry

IFN response in HeLa-TI-ISRE-mCherry cells treated with PSMs was assessed by the proportion of the cells expressing mCherry driven by ISRE as well as by mCherry mean fluorescence intensity (MFI) using a BD FACSCanto™ II flow cytometer (BD Biosciences, San Jose, CA, USA). Cells were seeded in 6-well plates (10^5 cells per well in 2 ml DMEM) and incubated with PSM at non-toxic concentrations for 24 h. For PSMs inducing IFN response after 24 h treatment we studied the dynamics of their effects at 1, 6 and 24 h. After the treatment with PSMs cells were removed from the culture plates using Versene Solution and 0.25% trypsin-EDTA and washed with PBS. To maintain high cell viability, a PBS solution with 2% fetal bovine serum was used as a cell storage buffer. The concentration of dimethyl sulfoxide (DMSO) in the medium for all compounds did not exceed 0.01%. All experiments were performed in triplicate biological replicates. The obtained data were analyzed using WinList™ 3D software (Version 9.0.1, Verity Software House, https://www.vsh.com/products/winlist/index.asp, Topsham, ME, USA).

4.6. Analysis of PSM induced LINE1 activation by immunofluorescent antibody staining and flow cytometry

To analyze PSM induced LINE1 activation, HeLa cells were seeded in 6-well plates (10^5 cells per well in 2 ml DMEM). After 24 h, cells were treated with compounds of interest at IC20 or non-toxic concentrations and incubated for 24/72 h. Then, the cells were removed from the substrate with trypsin, washed three times with PBS and fixed in cold 4% paraformaldehyde for 15 min. After the next three washes with PBS, they were permeabilized with cold 0.3% Triton-X100 for 7 minutes and blocked with bovine serum albumin for 1 h. Cells were immunofluorescently stained with antibodies to LINE1-ORF1, γ-H2AX, and subsequent binding with secondary antibodies AlexaFluor488 was carried

out in the dark. Cells were washed with PBS and analyzed on a BD FACSCanto™ II flow cytometer (BD Biosciences, San Jose, CA, USA). Proportions of the cells positive for the fluorescent signal and the average intensity of the cell fluorescence normalized to the control were assessed. The obtained data were analyzed using WinList™ 3D software (Version 9.0.1, Verity Software House, https://www.vsh.com/products/winlist/index.asp, Topsham, ME, USA).

4.7. Annexin-FITC/Propidium Iodide Double Staining

Cells were stained with annexin V-FITC and PI to evaluate apoptosis by flow cytometry according to the manufacturer's instructions to the FITC Anexin V Apoptosis Detection Kit I (Sigma-Aldrich, St. Louis, MI, USA). Cells were treated with maximum non-toxic concentrations of PSM for 24 h. After treatment, cells were collected, washed twice with ice-cold PBS, and resuspended in 0.5 mL of annexin/V-FITC/PI solution for 30 min in the dark according to manufacturer protocol. After staining at room temperature, cells were analyzed by the BD FACSCanto™ II flow cytometer (BD Biosciences, San Jose, CA, USA). For each sample, 10,000 events were acquired and positive FITC and/or PI cells were quantified using WinList™ 3D software (Version 9.0.1, Verity Software House, https://www.vsh.com/products/winlist/index.asp, Topsham, ME, USA).

4.8. Statistical Analysis

We compared the data from the experimental and control groups using one-way analysis of variance (ANOVA) and Dunnett's post hoc test. Differences between groups were considered significant at a p-value <0.05. The basis for statistical processing of results to determine the presence of statistically significant differences between several groups for one independent variable is the randomness of the samples, the equality of the sample size and the normality of the distributions of the samples used. The normality of data distribution was assessed with the Kolmogorov–Smirnov test. Statistical analyses were performed using GraphPad Prism 8.3.0 (GraphPad Software Inc., San Diego, CA, USA).

5. Conclusions

PSMs are important chemical components of the plants, actively used in human nutrition. Their active use may be explained by PSMs influence on human health and is considered to be a result of coevolution of flora and fauna. Our study revealed that linker histone H1.2 eviction from chromatin, caused by a number of DNA-binding anticancer natural small molecules known as PSMs, is accompanied by their activating influence on type I IFN signaling. In contrast, PSMs, not influencing linker histone H1.2 locations in nucleus, do not change type I IFN signaling activity. It let us propose a new mechanism of type I IFN signaling activation by environmental DNA-binding small molecules presented by PSMs, which cause chromatin destabilization. It is in agreement with "the Danger theory" proposed by P. Matzinger, however, it requires additional studies to elucidate damage associated molecular patterns formed after PSM-DNA interaction, as well as their PRRs and peculiar gene targets of activated PRRs that represent a new field of PSM investigations. Moreover, further studies of various PSMs with rather similar as well as different chemical structures in comparison with modeling data of their binding to different DNA motives and their effects on the locations of various linker histones will reveal PSM structural peculiarities activating type I IFN response, which enables anticancer immunity. Also, in further studies, it would be interesting to expand the range of model cell lines of various histogenesis and to assess the contribution of interferon signaling activation by the studied compounds to the anti-tumor effect *in vivo*. These data are important for elaboration of anticancer drugs of a new type, which are chromatin destabilizers activating antitumor immunity.

Supplementary Materials: Supplementary Material_1 (Table S1, Figure S1), Supplementary Material_2 (Table S2).

List of abbreviations

ANOVA – Analysis of Variance

DAMPs – Danger-associated Molecular Patterns

EGCG – Epigallocatechin-3-gallate

HSTPF – Human Signal Transduction Pathway Finder

IFN – Interferon

ISRE – Interferon-sensitive Response Element

L1– Long Interspersed Element-1

LINEs (L1) ,–Long Interspersed Nuclear Elements

LPS – Lipopolysaccharide

MFI – Mean Fluorescence Intensity

NLR – Nod-like-Receptor

PRRs – Pattern Recognition Receptors

PSMs – Plant Secondary Metabolites

RIG-1 – Retinoic Acid-inducible Gene 1

SCID – Severe Combined Immunodeficiency

SINEs – Short Interspersed Nuclear Elements

SVA – SINE-VNTR-Alus

TEs – Transposable Elements

TLR – Toll-like Receptor

Author Contributions: Conceptualization, O.V. and M.Y.; methodology, P.Sh., T.Z. and A.B..; software, A.B..; validation, A.O..; formal analysis, I.A., Kh.M., A.O. and P.Sh.; investigation, O.V., I.A., Kh.M. and P.Sh.; resources, I.A.; writing—original draft preparation, O.V. and M.Y.; writing—review and editing, G.B. and K.K..; visualization, O.V. and M.Y.; supervision, A.J. and I.B..; project administration, G.B. All authors have read and agreed to the published version of the manuscript.

Funding: The research was supported by the Russian Science Foundation grant 23-25-00276.

Institutional Review Board Statement: Not applicable.

Informed Consent Statement: Not applicable.

Data Availability Statement: Data presented in this study are contained within this article and in the supplementary materials, or are available upon request to the corresponding author.

Acknowledgments: We would like to thank Professor K. Gurova for providing us the plasmid with ISRE construction and valuable comments during the manuscript discussions, V. Nurtdinova for cell culturing before experiments and I. Dronova for help in the manuscript editing.

Conflicts of Interest: The authors declare no conflicts of interest.

References

1. Barnes, S. Effect of genistein on in vitro and in vivo models of cancer. The Journal of Nutrition. 1995. 125, 3 Suppl, 777S-783S. DOI:https://doi.org/10.1093/jn/125.3_Suppl.777S.
2. Bishayee, A. Cancer prevention and treatment with resveratrol: from rodent studies to clinical trials. Cancer Prevention Research (Philadelphia, Pa.). 2009. 409–418. DOI:https://doi.org/10.1158/1940-6207.CAPR-08-0160.

3. Kisková, T., Ekmekcioglu, C., Garajová, M. et al. A combination of resveratrol and melatonin exerts chemopreventive effects in N-methyl-N-nitrosourea-induced rat mammary carcinogenesis. European journal of cancer prevention: the official journal of the European Cancer Prevention Organisation (ECP). 2012. 21, 2, 163–170. DOI:https://doi.org/10.1097/CEJ.0b013e32834c9c0f.
4. Whitsett, T. G. and Lamartiniere, C. A. Genistein and resveratrol: mammary cancer chemoprevention and mechanisms of action in the rat. Expert Review of Anticancer Therapy. 2006. 6, 12, 1699–1706. DOI:https://doi.org/10.1586/14737140.6.12.1699.
5. Fantini, M., Benvenuto, M., Masuelli, L. et al. In Vitro and in Vivo Antitumoral Effects of Combinations of Polyphenols, or Polyphenols and Anticancer Drugs: Perspectives on Cancer Treatment. International Journal of Molecular Sciences. 2015. 16, 5, 9236–9282. DOI:https://doi.org/10.3390/ijms16059236.
6. Jantan, I., Ahmad, W. and Bukhari, S. N. A. Plant-derived immunomodulators: an insight on their preclinical evaluation and clinical trials. Frontiers in Plant Science. 2015. 6, 655. DOI:https://doi.org/10.3389/fpls.2015.00655.
7. Pezzuto, J. M. Resveratrol: Twenty Years of Growth, Development and Controversy. Biomolecules & Therapeutics. 2019. 27, 1, 1–14. DOI:https://doi.org/10.4062/biomolther.2018.176.
8. Russo, M., Russo, G. L., Daglia, M. et al. Understanding genistein in cancer: The "good" and the "bad" effects: A review. Food Chemistry. 2016. 196, 589–600. DOI:https://doi.org/10.1016/j.foodchem.2015.09.085.
9. Chen, Y.-X., Gao, Q.-Y., Zou, T.-H. et al. Berberine versus placebo for the prevention of recurrence of colorectal adenoma: a multicentre, double-blinded, randomised controlled study. The Lancet Gastroenterology & Hepatology. 2020. 5, 3, 267–275. DOI:https://doi.org/10.1016/S2468-1253(19)30409-1.
10. Thomas, R., Williams, M., Sharma, H. et al. A double-blind, placebo-controlled randomised trial evaluating the effect of a polyphenol-rich whole food supplement on PSA progression in men with prostate cancer—the UK NCRN Pomi-T study. Prostate Cancer and Prostatic Diseases. 2014. 17, 2, 180–186. DOI:https://doi.org/10.1038/pcan.2014.6.
11. Zhang, H., Gordon, R., Li, W. et al. Genistein treatment duration effects biomarkers of cell motility in human prostate. PLOS ONE. 2019. 14, 3, e0214078. DOI:https://doi.org/10.1371/journal.pone.0214078.
12. Britton, R. G. Kovoor, C. and Brown, K. Direct molecular targets of resveratrol: identifying key interactions to unlock complex mechanisms: Direct molecular targets of resveratrol. Annals of the New York Academy of Sciences. 2015. 1348, 1, 124–133. DOI:https://doi.org/10.1111/nyas.12796.
13. Khan, F., Niaz, K., Maqbool, F. et al. Molecular Targets Underlying the Anticancer Effects of Quercetin: An Update. Nutrients. 2016. 8, 9, 529. DOI:https://doi.org/10.3390/nu8090529.
14. Nagaraju, G. P. Zafar, S. F. and El-Rayes, B. F. Pleiotropic effects of genistein in metabolic, inflammatory, and malignant diseases. Nutrition Reviews. 2013. 71, 8, 562–572. DOI:https://doi.org/10.1111/nure.12044.
15. Qadir, M. I., Naqvi, S. T. Q. and Muhammad, S. A. Curcumin: a Polyphenol with Molecular Targets for Cancer Control. Asian Pacific journal of cancer prevention: APJCP. 2016. 17, 6, 2735–2739.
16. N'soukpoé-Kossi, C. N., Bourassa, P., Mandeville, J. S. et al. Structural modeling for DNA binding to antioxidants resveratrol, genistein and curcumin. Journal of Photochemistry and Photobiology B: Biology. 2015. 151, 69–75. DOI:https://doi.org/10.1016/j.jphotobiol.2015.07.007.
17. Kanakis, C. D., Tarantilis, P. A., Polissiou, M. G. and Tajmir-Riahi, H.-A. Interaction of Antioxidant Flavonoids with tRNA: Intercalation or External Binding and Comparison with Flavonoid-DNA Adducts. DNA and Cell Biology. 2006. 25, 2, 116–123. DOI:https://doi.org/10.1089/dna.2006.25.116.
18. Nafisi, S. Hashemi, M., Rajabi, M. and Tajmir-Riahi, H. A. DNA Adducts with Antioxidant Flavonoids: Morin, Apigenin, and Naringin. DNA and Cell Biology. 2008. 27, 8, 433–442. DOI:https://doi.org/10.1089/dna.2008.0735.
19. Bhattacharjee, S. Chakraborty, S., Sengupta, P. K. and Bhowmik, S. Exploring the Interactions of the Dietary Plant Flavonoids Fisetin and Naringenin with G-Quadruplex and Duplex DNA, Showing Contrasting Binding Behavior: Spectroscopic and Molecular Modeling Approaches. The Journal of Physical Chemistry. B. 2016. 120, 34, 8942–8952. DOI:https://doi.org/10.1021/acs.jpcb.6b06357.
20. Galindo-Murillo, R. and Cheatham, T. E. Computational DNA binding studies of (–)-epigallocatechin-3-gallate. Journal of Biomolecular Structure and Dynamics. 2018. 36, 13, 3311–3323. DOI:https://doi.org/10.1080/07391102.2017.1389306.
21. Khurana, S. Kukreti, S. and Kaushik, M. Designing a two-stage colorimetric sensing strategy based on citrate reduced gold nanoparticles: Sequential detection of Sanguinarine (anticancer drug) and visual sensing of DNA. Spectrochimica Acta Part A: Molecular and Biomolecular Spectroscopy. 2021. 246, 119039. DOI:https://doi.org/10.1016/j.saa.2020.119039.
22. Basu, A. and Kumar, G. S. Biophysical studies on curcumin–deoxyribonucleic acid interaction: Spectroscopic and calorimetric approach. International Journal of Biological Macromolecules. 2013. 62, 257–264. DOI:https://doi.org/10.1016/j.ijbiomac.2013.09.003.
23. Pandya, N. Khan, E., Jain, N. et al. Curcumin analogs exhibit anti-cancer activity by selectively targeting G-quadruplex forming c-myc promoter sequence. Biochimie. 2021. 180, 205–221. DOI:https://doi.org/10.1016/j.biochi.2020.11.006.
24. Mikutis, G., Karaköse, H., Jaiswal, R. et al. Phenolic promiscuity in the cell nucleus--epigallocatechingallate (EGCG) and theaflavin-3,3′-digallate from green and black tea bind to model cell nuclear structures including histone proteins, double stranded DNA and telomeric quadruplex DNA. Food & Function. 2013. 4, 2, 328–337. DOI:https://doi.org/10.1039/c2fo30159h.
25. Bhattacharjee, S., Chakraborty, S., Chorell, E. et al. Importance of the hydroxyl substituents in the B–ring of plant flavonols on their preferential binding interactions with VEGF G–quadruplex DNA: Multi-spectroscopic and molecular modeling studies. International Journal of Biological Macromolecules. 2018. 118, 629–639. DOI:https://doi.org/10.1016/j.ijbiomac.2018.06.115.

26. Dickerhoff, J., Brundridge, N., McLuckey, S. A. and Yang, D. Berberine Molecular Recognition of the Parallel MYC G-Quadruplex in Solution. Journal of medicinal chemistry. 2021. 64, 21, 16205–16212. DOI:https://doi.org/10.1021/acs.jmedchem.1c01508.
27. Jarosova, P., Paroulek, P., Rajecky, M. et al. Naturally occurring quaternary benzo[c]phenanthridine alkaloids selectively stabilize G-quadruplexes. Physical chemistry chemical physics: PCCP. 2018. 20, 33, 21772–21782. DOI:https://doi.org/10.1039/c8cp02681e.
28. Tawani, A. Mishra, S. K. and Kumar, A. Structural insight for the recognition of G-quadruplex structure at human c-myc promoter sequence by flavonoid Quercetin. Scientific Reports. 2017. 7, 3600. DOI:https://doi.org/10.1038/s41598-017-03906-3.
29. Salem, A. A., El Haty, I. A., Abdou, I. M. and Mu, Y. Interaction of human telomeric G-quadruplex DNA with thymoquinone: A possible mechanism for thymoquinone anticancer effect. Biochimica et Biophysica Acta (BBA) - General Subjects. 2015. 1850, 2, 329–342. DOI:https://doi.org/10.1016/j.bbagen.2014.10.018.
30. Luzhin, A., Rajan, P., Safina, A. et al. Comparison of cell response to chromatin and DNA damage. Nucleic Acids Res. 2023; 51(21):11836-11855. doi: 10.1093/nar/gkad865. Erratum in: Nucleic Acids Res. 2024; 52(2):999. doi: 10.1093/nar/gkad1192.
31. Kirsanov, K.I., Kotova, E., Makhov, P. et al. Minor grove binding ligands disrupt PARP-1 activation pathways. Oncotarget. 2014; 5(2):428-37. doi: 10.18632/oncotarget.1742.
32. Vlasova, O., Antonova, I., Zenkov, R. et al. Anticancer Plant Secondary Metabolites Induce Linker Histone Depletion from Chromatin. Frontiers in Bioscience-Landmark, 2024, 29(7) https://doi.org/10.31083/j.fbl23690.
33. Izquierdo-Bouldstridge, A., Bustillos, A., Bonet-Costa, C. et al. Histone H1 depletion triggers an interferon response in cancer cells via activation of heterochromatic repeats. Nucleic Acids Research. 2017. 45, 20, 11622. DOI: 10.1093/nar/gkx746.
34. Salinas-Pena M, Serna-Pujol N, Jordan A. Genomic profiling of six human somatic histone H1 variants denotes that H1X accumulates at recently incorporated transposable elements. Nucleic Acids Res. 2024 Feb 28;52(4):1793-1813. doi: 10.1093/nar/gkae014.
35. Healton, S. E., Pinto, H. D., Mishra, L. N. et al. H1 linker histones silence repetitive elements by promoting both histone H3K9 methylation and chromatin compaction. Proc Natl Acad Sci USA. 2020. 117(25):14251-14258. DOI: 10.1073/pnas.1920725117.
36. Pang, B., Qiao, X., Janssen, L. et al. Drug-induced histone eviction from open chromatin contributes to the chemotherapeutic effects of doxorubicin. Nat Commun. 2013; 4:1908. doi: 10.1038/ncomms2921.
37. Safina, A., Cheney, P., Pal, M. et al. FACT is a sensor of DNA torsional stress in eukaryotic cells. Nucleic Acids Res. 2017; 45(4):1925-1945. doi: 10.1093/nar/gkw1366.
38. Leonova, K., Safina, A., Nesher, E. et al. TRAIN (Transcription of Repeats Activates INterferon) in response to chromatin destabilization induced by small molecules in mammalian cells. Elife. 2018. 7:e30842. DOI: 10.7554/eLife.30842.
39. Chen, M., Brackett, C.M., Burdelya, L.G. et al. Stimulation of an anti-tumor immune response with "chromatin-damaging" therapy. Cancer Immunol Immunother. 2021; 70(7):2073-2086. doi: 10.1007/s00262-020-02846-8.
40. Stilp, A. C., Scherer, M., König, P., Fürstberger, A. et al. The chromatin remodeling protein ATRX positively regulates IRF3-dependent type I interferon production and interferon-induced gene expression. PLoS Pathog. 2022. 18(8):e1010748. DOI: 10.1371/journal.ppat.1010748.
41. Platanias, L.C. Mechanisms of type-I- and type-II-interferon-mediated signalling. Nat Rev Immunol. 2005. 5(5):375-86. DOI: 10.1038/nri1604.
42. Petrova, L., Bunz, F. Interferons in Colorectal Cancer Pathogenesis and Therapy. Dis Res. 2024. 4(1):31-39. DOI: 10.54457/dr.202401005.
43. Jiao, F. and Gong, Z. The Beneficial Roles of SIRT1 in Neuroinflammation-Related Diseases. Oxidative Medicine and Cellular Longevity. 2020. 6782872. DOI:https://doi.org/10.1155/2020/6782872.
44. Alesci, A., Nicosia, N., Fumia, A. et al. Resveratrol and Immune Cells: A Link to Improve Human Health. Molecules. 2022. 27, 2, 424. DOI:https://doi.org/10.3390/molecules27020424.
45. Sun, J., Zeng, Q., Wu, Z. et al. Berberine inhibits NLRP3 inflammasome activation and proinflammatory macrophage M1 polarization to accelerate peripheral nerve regeneration. Neurotherapeutics. 2024. 21, 4, e00347. DOI:https://doi.org/10.1016/j.neurot.2024.e00347.
46. Lani, R., Teoh, B.-T., Sam, S.-S. et al. Fisetin Modulates Toll-like Receptor-Mediated Innate Antiviral Response in Chikungunya Virus-Infected Hepatocellular Carcinoma Huh7 Cells. Immuno. 2022. 2, 4, 703–719. DOI:https://doi.org/10.3390/immuno2040043.
47. Kataoka, H., Saeki, A., Hasebe, A. et al. Naringenin suppresses Toll-like receptor 2-mediated inflammatory responses through inhibition of receptor clustering on lipid rafts. Food Science & Nutrition. 2020. 9, 2, 963–972. DOI:https://doi.org/10.1002/fsn3.2063.
48. Yu, J., Shi, H., Song, K. et al. Naringenin Improves Innate Immune Suppression after PRRSV Infection by Reactivating the RIG-I-MAVS Signaling Pathway, Promoting the Production of IFN-I. Viruses. 2023. 15, 11, 2172. DOI:https://doi.org/10.3390/v15112172.
49. Sunthamala, N., Suebsamran, C., Khruaphet, N. et al. Sanguinarine and Chelidonine Synergistically Induce Endosomal Toll-like Receptor and M1-Associated Mediators Expression. Journal of Pure and Applied Microbiology. 2020. 14(4):2351-2361. DOI:10.22207/JPAM.14.4.13
50. Bhaskar, S. and Helen, A. Quercetin modulates toll-like receptor-mediated protein kinase signaling pathways in oxLDL-challenged human PBMCs and regulates TLR-activated atherosclerotic inflammation in hypercholesterolemic rats. Molecular and Cellular Biochemistry. 2016. 423, 1–2, 53–65. DOI:https://doi.org/10.1007/s11010-016-2824-9.

51. Warowicka, A., Nawrot, R. and Goździcka-Józefiak, A. Antiviral activity of berberine. Archives of Virology. 2020. 165, 9, 1935–1945. DOI:https://doi.org/10.1007/s00705-020-04706-3.
52. De Cecco, M., Ito, T., Petrashen, A. P., Elias, A.E. et al. L1 drives IFN in senescent cells and promotes age-associated inflammation. Nature. 2019. 566(7742):73-78. DOI: 10.1038/s41586-018-0784-9.
53. Zenkov, R. G., Kirsanov, K. I., Ogloblina, A. M., Vlasova, O. A. et al. Effects of G-Quadruplex-Binding Plant Secondary Metabolites on c-MYC Expression. Int J Mol Sci. 2022. 23(16):9209. doi: 10.3390/ijms23169209.
54. Amarante-Mendes, G. P., Adjemian, S., Branco, L. M. et al. Pattern Recognition Receptors and the Host Cell Death Molecular Machinery. Front Immunol. 2018, 9, 2379. DOI: 10.3389/fimmu.2018.02379.
55. Carroll, S.L., Pasare, C., Barton, G.M. Control of adaptive immunity by pattern recognition receptors. Immunity. 2024; 57(4):632-648. doi: 10.1016/j.immuni.2024.03.014.
56. Jiao, F., Gong, Z. The Beneficial Roles of SIRT1 in Neuroinflammation-Related Diseases. Oxid Med Cell Longev. 2020. 2020:6782872. DOI: 10.1155/2020/6782872.
57. Silverstein, A. M. Paul Ehrlich's passion: the origins of his receptor immunology. Cell Immunol. 1999. 194(2):213-21. DOI: 10.1006/cimm.1999.1505.
58. Matzinger, P. Tolerance, danger, and the extended family. Annu Rev Immunol. 1994. 12:991-1045. DOI: 10.1146/annurev.iy.12.040194.005015.
59. Matzinger, P. The danger model: a renewed sense of self. Science. 2002. 296(5566):301-5. DOI: 10.1126/science.1071059.
60. Ancona, G., Alagna, L., Alteri, C. et al. Gut and airway microbiota dysbiosis and their role in COVID-19 and long-COVID. Front Immunol. 2023 Mar 8;14:1080043. DOI: 10.3389/fimmu.2023.1080043.
61. Stetson, D. B., Medzhitov, R. Type I interferons in host defense. Immunity. 2006. 25(3):373-81. DOI: 10.1016/j.immuni.2006.08.007.
62. Seo, D. J., Choi, C. Inhibitory mechanism of five natural flavonoids against murine norovirus. Phytomedicine. 2017 Jul. 30:59-66. DOI: 10.1016/j.phymed.2017.04.011.
63. Lin, C. J., Lin, H. J., Chen, T.H. et al. Polygonum cuspidatum and its active components inhibit replication of the influenza virus through toll-like receptor 9-induced interferon beta expression. PLoS One. 2015. 6;10(2):e0117602. DOI: 10.1371/journal.pone.0117602.
64. Kim, J. H., Weeratunga, P., Kim, M. S. et al. Inhibitory effects of an aqueous extract from Cortex Phellodendri on the growth and replication of broad-spectrum of viruses in vitro and in vivo. BMC Complement Altern Med. 2016. 16:265. doi: 10.1186/s12906-016-1206-x.
65. Fast, D. J., Stern, N. P., Chuang, J. et al. Flavanones common to citrus fruits activate the interferon-stimulated response element by stimulating expression of IRF7. Journal of Food Bioactives. 2019. 8. DOI: 10.31665/JFB.2019.8207.
66. Akash, S., Bayil, I., Rahman, Md A. et al. Target specific inhibition of West Nile virus envelope glycoprotein and methyltransferase using phytocompounds: an in silico strategy leveraging molecular docking and dynamics simulation. Front. Microbiol. 2023. 14. DOI: 10.3389/fmicb.2023.1189786.
67. Ullah, T. R., Balka, K. R., Ambrose, R. L et al. Genistein Targets STING-Driven Antiviral Responses. mBio. 2022. 13(4):e0206422. DOI: 10.1128/mbio.02064-22.
68. LeCher, J. C, Diep, N., Krug, P. W., Hilliard, J. K. Genistein Has Antiviral Activity against Herpes B Virus and Acts Synergistically with Antiviral Treatments to Reduce Effective Dose. Viruses. 2019. 11(6):499. DOI: 10.3390/v11060499.
69. Aziz, N., Son, Y. J., Cho, J. Y. Thymoquinone Suppresses IRF-3-Mediated Expression of Type I Interferons via Suppression of TBK1. Int J Mol Sci. 2018. 19(5):1355. DOI: 10.3390/ijms19051355.
70. Lee, M. H., Seo, D. J., Kang, J. H. et al. Expression of antiviral cytokines in Crandell-Reese feline kidney cells pretreated with Korean red ginseng extract or ginsenosides. Food Chem Toxicol. 2014. 70:19-25. DOI: 10.1016/j.fct.2014.04.034.
71. Li, S., Yang, L. J., Wang, P. et al. Dietary apigenin potentiates the inhibitory effect of interferon-α on cancer cell viability through inhibition of 26S proteasome-mediated interferon receptor degradation. Food Nutr Res. 2016. 60:31288. DOI: 10.3402/fnr.v60.31288.
72. Volkman, H. E., Stetson, D. B. The enemy within: endogenous retroelements and autoimmune disease. Nat Immunol. 2014. 15(5):415-22. DOI: 10.1038/ni.2872.
73. Molagoda, I. M. N., Athapaththu, A. M. G. K, Choi, Y. H. et al. Fisetin Inhibits NLRP3 Inflammasome by Suppressing TLR4/MD2-Mediated Mitochondrial ROS Production. Antioxidants (Basel). 2021. 10(8):1215. DOI: 10.3390/antiox10081215.
74. Jiang, K., Yang, J., Xue, G. et al. Fisetin Ameliorates the Inflammation and Oxidative Stress in Lipopolysaccharide-Induced Endometritis. J Inflamm Res. 2021. 14:2963-2978. DOI: 10.2147/JIR.S314130.
75. Huang, X., Shen, H., Liu, Y. et al. Fisetin attenuates periodontitis through FGFR1/TLR4/NLRP3 inflammasome pathway. Int Immunopharmacol. 2021. 95:107505. DOI: 10.1016/j.intimp.2021.107505.
76. Lani, R., Teoh, B.-T., Sam, S.-S. et al. Fisetin Modulates Toll-like Receptor-Mediated Innate Antiviral Response in Chikungunya Virus-Infected Hepatocellular Carcinoma Huh7 Cells. Immuno. 2022. 2: 703-719. DOI: 10.3390/immuno2040043.
77. Domiciano, T. P., Wakita, D., Jones, H.D. et al. Quercetin Inhibits Inflammasome Activation by Interfering with ASC Oligomerization and Prevents Interleukin-1 Mediated Mouse Vasculitis. Sci Rep. 2017. 7:41539. DOI: 10.1038/srep41539.
78. Alattar, A., Alshaman, R., Althobaiti, Y. S. et al. Quercetin Alleviated Inflammasome-Mediated Pyroptosis and Modulated the mTOR/P70S6/P6/eIF4E/4EBP1 Pathway in Ischemic Stroke. Pharmaceuticals (Basel). 2023. 16(8):1182. DOI: 10.3390/ph16081182.
79. Malaguarnera, L. Influence of Resveratrol on the Immune Response. Nutrients. 2019. 11(5):946. DOI: 10.3390/nu11050946.

80. Wang, B., Bellot, G. L., Iskandar, K. et al. Resveratrol attenuates TLR-4 mediated inflammation and elicits therapeutic potential in models of sepsis. Sci Rep. 2020. 10(1):18837. DOI: 10.1038/s41598-020-74578-9.
81. Jeong, J. W., Lee, H. H., Han, M. H. et al. Anti-inflammatory effects of genistein via suppression of the toll-like receptor 4-mediated signaling pathway in lipopolysaccharide-stimulated BV2 microglia. Chem Biol Interact. 2014. 212:30-9. DOI: 10.1016/j.cbi.2014.01.012.
82. Bae, C. H., Jeon, B. S., Choi, Y. S. et al. Delphinidin Inhibits LPS-Induced MUC8 and MUC5B Expression Through Toll-like Receptor 4-Mediated ERK1/2 and p38 MAPK in Human Airway Epithelial Cells. Clin Exp Otorhinolaryngol. 2014. 7(3):198-204. DOI: 10.3342/ceo.2014.7.3.198.
83. Shen, X. Y., Liu, X. P., Song, C. K. et al. Genome-wide analysis reveals alcohol dehydrogenase 1C and secreted phosphoprotein 1 for prognostic biomarkers in lung adenocarcinoma. J Cell Physiol. 2019. 234(12):22311-22320. DOI: 10.1002/jcp.28797.
84. Chang, S., Li, X., Zheng, Y. et al. Kaempferol exerts a neuroprotective effect to reduce neuropathic pain through TLR4/NF-κB signaling pathway. Phytother Res. 2022. 36(4):1678-1691. DOI: 10.1002/ptr.7396.
85. Meng, Y. Y., Liu, Y., Hu, Z. F. et al. Sanguinarine Attenuates Lipopolysaccharide-induced Inflammation and Apoptosis by Inhibiting the TLR4/NF-κB Pathway in H9c2 Cardiomyocytes. Curr Med Sci. 2018. 38(2):204-211. DOI 10.1007/s11596-018-1867-4.
86. Sunthamala, N., Suebsamran, C., Khruaphet, N. et al. Sanguinarine and Chelidonine synergistically induce endosomal toll-like receptor and M1-associated mediators expression. J. Pure Appl. Microbiol. 2020. 14, 2351–2361. DOI: 10.22207/JPAM.14.4.13.
87. Bao, S., Cao, Y., Zhou, H. et al. Epigallocatechin gallate (EGCG) suppresses lipopolysaccharide-induced Toll-like receptor 4 (TLR4) activity via 67 kDa laminin receptor (67LR) in 3T3-L1 adipocytes. J Agric Food Chem. 2015. 63(10):2811-9. DOI: 10.1021/jf505531w.
88. Chen, B., Li, Y.F., Fang, Z. et al. Epigallocatechin-3-gallate protects sepsis-induced myocardial dysfunction by inhibiting the nuclear factor-κB signaling pathway. Heliyon. 2024; 10(5):e27163. doi: 10.1016/j.heliyon.2024.e27163.
89. Kim, A., Lee, C. S. Apigenin reduces the Toll-like receptor-4-dependent activation of NF-κB by suppressing the Akt, mTOR, JNK, and p38-MAPK. Naunyn Schmiedebergs Arch Pharmacol. 2018. 391(3):271-283. DOI: 10.1007/s00210-017-1454-4.
90. Xiao, S., Zhang, P., Zhang, G. et al. Inhibition of toll-like receptor 4 activation by apigenin and chrysin via competition for sites and conformational changes. Int J Biol Macromol. 2023; 252:126415. doi: 10.1016/j.ijbiomac.2023.126415.
91. Zhao, B., Zhang, S., Amin, N. et al. Thymoquinone regulates microglial M1/M2 polarization after cerebral ischemia-reperfusion injury via the TLR4 signaling pathway. Neurotoxicology. 2024. 101:54-67. DOI: 10.1016/j.neuro.2024.02.002.
92. Gao, H., Kang, N., Hu, C. et al. Ginsenoside Rb1 exerts anti-inflammatory effects in vitro and in vivo by modulating toll-like receptor 4 dimerization and NF-kB/MAPKs signaling pathways. Phytomedicine. 2020. 69:153197. DOI: 10.1016/j.phymed.2020.153197.
93. Rostom, B., Karaky, R., Kassab, I., Sylla-Iyarreta Veitía, M. Coumarins derivatives and inflammation: Review of their effects on the inflammatory signaling pathways. Eur J Pharmacol. 2022. 922:174867. DOI: 10.1016/j.ejphar.2022.174867.

Disclaimer/Publisher's Note: The statements, opinions and data contained in all publications are solely those of the individual author(s) and contributor(s) and not of MDPI and/or the editor(s). MDPI and/or the editor(s) disclaim responsibility for any injury to people or property resulting from any ideas, methods, instructions or products referred to in the content.
