# Supplementary material for: Anticancer Plant Secondary Metabolites Evicting Linker Histone H1.2 from Chromatin Activate Type I Interferon Signaling
Source: Int J Mol Sci. 2025 Jan 4;26(1):375. doi: 10.3390/ijms26010375 (PMC11722331; doi:10.3390/ijms26010375)

Table S1.

Fluorescence and cytotoxicity data of the PSMs. For PSM IC50 and IC20 values ​​and non-toxic concentrations were obtained from the approximate logistic function of the resorufin fluorescence intensity at 592 nm. Maximum non-toxic concentrations were confirmed by PI/annexin staining of cells and subsequent flow cytometric analysis.

| **Title** | **Fluorescence** | | **Cytotoxicity of PSMs against HeLa cells after 24-hour exposure** | | |
| --- | --- | --- | --- | --- | --- |
|  | **Excitation/Emission spectra, nm** | **Effect on fluorescence in experiment for mCherry** | **IC50 (µM)** | **IC20 (µM)** | **IC0 (µM)** |
| Fisetin | 280/567  High level | At concentration > IC20 | 553  ±  45 | 118  ±  13 | 27 |
| Quercetin | no | no | 109  ±  6 | 34  ±  3 | 10 |
| Resveratrol | 319/400  High level | no | 707  ±  57 | 233  ±  24 | 50 |
| Berberine | 458/550  Low level | At concentration > IC50 | 709  ±  48 | 108  ±  11 | 10 |
| Genistein | no | no | 842  ±  43 | 176  ±  18 | 60 |
| Naringenin | no | no | 457  ±  17 | 236  ±  11 | 52 |
| Delphinidin | 310/381  Low level | no | 874  ±  78 | 284  ±  34 | 100 |
| Curcumin | no | no | 32  ±  2 | 15  ±  1 | 7.5 |
| Kaempferol | no | no | 115  ±  9 | 10  ±  2 | 2 |
| Sanguinarine | 480/600  High level | At concentration > IC20 | 4.0  ±  0.2 | 2.1  ±  0.2 | 0.8 |
| EGCG | 328/391  Low level | no | 282  ±  28 | 133  ±  20 | 65 |
| Coumarin | no | no | 1868  ±  98 | 860  ±  66 | 260 |
| Ginsenoside Rb1 | no | no | 285  ±  22 | 80  ±  4 | 30 |
| Thymoquinone | no | no | 33  ±  1 | 18  ±  1 | 3 |
| Apigenin | no | no | 52  ±  9 | 12  ±  2 | 5 |

Figure S1. Plant secondary metabolites: structural formulae and influence on histone H1.2 and H1.4 depletion.
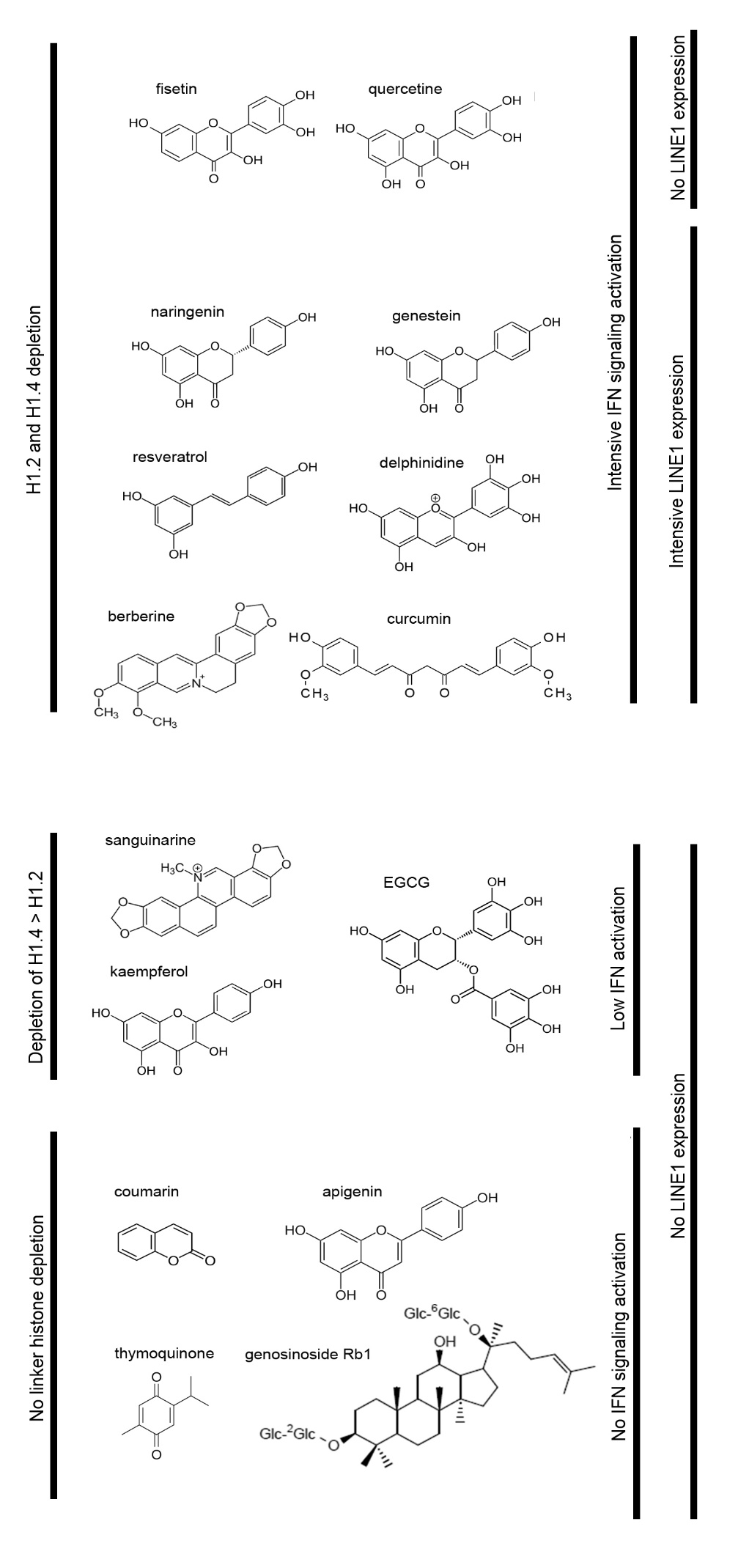

Supplement: Supplementary file 1 [file ijms-26-00375-s001.zip › Supplementary Material_1.docx]
